# Sulfonylureas exert antidiabetic action on adipocytes by inhibition of PPARγ serine 273 phosphorylation

**DOI:** 10.1016/j.molmet.2024.101956

**Published:** 2024-05-10

**Authors:** Bodo Haas, Moritz David Sebastian Hass, Alexander Voltz, Matthias Vogel, Julia Walther, Arijit Biswas, Daniela Hass, Alexander Pfeifer

**Affiliations:** 1 Federal Institute for Drugs and Medical Devices, Kurt-Georg-Kiesinger-Allee 3, Bonn, Germany; 2 MVZ für Hämostaseologie, Rheumathologie, Endokrinologie, Allgemeinmedizin und Transfusionsmedizin, München, Germany; 3 Institute of Pharmacology and Toxicology, University Hospital, University of Bonn, Bonn, Germany; 4 Institute of Experimental Hematology and Transfusion Medicine, University Hospital, University of Bonn, Bonn, Germany; 5 Institute for Diabetes and Cancer, Helmholtz Munich, German Center for Diabetes Research, Neuherberg, Germany

**Keywords:** Sulfonylureas, PPARγ, White adipose tissue, Brown adipose tissue

## Abstract

**Objective:**

Sulfonylureas (SUs) are still among the mostly prescribed antidiabetic drugs with an established mode of action: release of insulin from pancreatic β-cells. In addition, effects of SUs on adipocytes by activation of the nuclear receptor peroxisome proliferator-activated receptor γ (PPARγ) have been described, which might explain their insulin-sensitizing potential observed in patients. However, there is a discrepancy between the impact of SUs on antidiabetic action and their rather moderate *in vitro* effect on PPARγ transcriptional activity. Recent studies have shown that some PPARγ ligands can improve insulin sensitivity by blocking PPARγ Ser-273 phosphorylation without having full agonist activity. It is unknown if SUs elicit their antidiabetic effects on adipocytes by inhibition of PPARγ phosphorylation. Here, we investigated if binding of SUs to PPARγ can interfere with PPARγ Ser-273 phosphorylation and determined their antidiabetic actions *in vitro* in primary human white adipocytes and *in vivo* in high-fat diet (HFD) obese mice.

**Methods:**

Primary human white preadipocytes were differentiated in the presence of glibenclamide, glimepiride and PPARγ ligands rosiglitazone and SR1664 to compare PPARγ Ser-273 phosphorylation, glucose uptake and adipokine expression. Transcriptional activity at PPARγ was determined by luciferase assays, quantification of PPARγ Ser-273 phosphorylation was determined by Western blotting and CDK5 kinase assays*. In silico* modelling was performed to gain insight into the binding characteristics of SUs to PPARγ. HFD mice were administered SUs and rosiglitazone for 6 days. PPARγ Ser-273 phosphorylation in white adipose tissue (WAT), body composition, glucose tolerance, adipocyte morphology and expression levels of genes involved in PPARγ activity in WAT and brown adipose tissue (BAT) were evaluated.

**Results:**

SUs inhibit phosphorylation of PPARγ at Ser-273 in primary human white adipocytes and exhibit a positive antidiabetic expression profile, which is characterized by up regulation of insulin-sensitizing and down regulation of insulin resistance-inducing adipokines. We demonstrate that SUs directly bind to PPARγ by *in silico* modelling and inhibit phosphorylation in kinase assays to a similar extend as rosiglitazone and SR1664. In HFD mice SUs reduce PPARγ phosphorylation in WAT and have comparable effects on gene expression to rosiglitazone. In BAT SUs increase UCP1 expression and reduce lipid droplets sizes.

**Conclusions:**

Our findings indicate that a part of SUs extra-pancreatic effects on adipocytes *in vitro* and *in vivo* is probably mediated via their interference with PPARγ phosphorylation rather than via classical agonistic activity at clinical concentrations.

## Highlights

• Sulfonylureas (SUs) inhibit PPARγ serine 273 phosphorylation in primary human adipocytes and in adipose tissue of obese mice.
• SUs exhibit a positive antidiabetic expression profile in primary human adipocytes and obese mice.
• A new MoA of SUs is proposed which is mediated by inhibition of PPARγ phosphorylation rather than classical PPARγ agonism.

## 1 Introduction

Type 2 diabetes mellitus (T2DM) has reached pandemic dimensions with estimated 537 million affected patients worldwide [1]. First-line therapy includes lifestyle modifications and dietary adjustments. Nevertheless, pharmacotherapeutic intervention is crucial to control long-term consequences of T2DM. A hallmark of T2DM is insulin resistance of multiple organs such as liver, muscle and adipose tissue. Several antidiabetic medications are available which aim at correcting insulin resistance or to increase pancreatic insulin secretion [2]. Sulfonylureas (SUs) are a well-established class of antidiabetic drugs which are still extensively used and recommended for mono- or combination therapy in many parts of the world [3]. Their mode of action is stimulation of insulin secretion from pancreatic β-cells by binding to the sulfonylurea receptor 1 (SUR1) subunit of the adenosine triphosphate (ATP)-sensitive potassium channel on the plasma membrane [4]. Some pharmacodynamic effects of SUs such as improvement of insulin sensitivity or the ability to reduce circulating levels of pro-inflammatory cytokines while increasing levels of anti-inflammatory cytokines cannot be readily explained by increased insulin secretion [[5], [6], [7]]. It has previously been shown that SUs exert extra-pancreatic activity especially on white adipocytes. This includes induction of adipogenesis, increased glucose uptake and changes in adipokine expression profiles [[8], [9], [10], [11], [12], [13]]. The proposed mechanism of action is activation of the nuclear receptor peroxisome proliferator-activated receptor γ (PPARγ), a key regulator of adipocyte differentiation [14]. However, there is a discrepancy between the impact of SUs on antidiabetic action and their effect on PPARγ transcriptional activity. While antidiabetic effects are already measurable at nanomolar to low micromolar concentrations in primary human adipocytes [10], an increase of PPARγ promoter activity occurs in the higher micromolar range in *in vitro* experiments [8,12,13,15] and thus leaves the question whether all effects are indeed mediated by transcriptional activation of PPARγ at clinically relevant concentrations. PPARγ can be activated by endogenous ligands such as fatty acids and their metabolites, as well as by thiazolidinedions (TZDs) [16,17]. TZDs directly bind to the ligand-binding domain (LBD) of PPARγ, thereby activating the transcription of target genes, which are involved in many metabolic pathways. Due to their insulin-sensitizing effect, TZDs have been used to treat metabolic disorders such as T2DM, however the clinical use has subsequently declined in the last years owed to adverse effects [18]. Binding of ligands to PPARγ not only activates transcription but also blocks cyclin-dependent kinase 5 (CDK5) and/or extracellular signal-regulated kinase (ERK)-mediated phosphorylation of PPARγ at serine 273 (Ser-273). Phosphorylation of PPARγ by CDK5/ERK does not alter its adipogenic activity, but modifies expression of a specific set of genes with impact in obesity and diabetes [[19], [20], [21]]. Classical transcriptional activation of PPARγ, which mediates at least some of the undesirable side effects of chronic PPARγ activation, appears to be independent from Ser-273 phosphorylation. The exact mechanism is still not fully clear but it is suggested that selective co-regulator recruitment to PPARγ is regulated in a phosphorylation-dependent manner and controls expression of diabetes-related genes [22,23]. Prevention of PPARγ Ser-273 phosphorylation in adipose tissues and skeletal muscle protects mice from insulin resistance. This is associated with decreased levels of growth differentiation factor 3 (GDF3), which is a member of the transforming growth factor β (TGF-β) superfamily that reduces insulin sensitivity by inhibition of bone morphogenic protein (BMP) signaling [24]. In addition, the protein phosphatase Mg2+/Mn2+-dependent 1A (PPM1A), which dephosphorylates Ser-273 of PPARγ can restore dysregulated genes involved in diabetes progression in obese mice [25]. Thus, PPARγ agonism is not necessarily correlated with antidiabetic action. Interestingly, PPARγ ligands which exert only low agonistic activity, like the experimental substance SR1664, are able to block PPARγ phosphorylation at Ser-273 and still have antidiabetic activity without inducing adverse effects such as fluid retention, bone fractures and weight gain in mice [20,26,27]. The inhibition of PPARγ phosphorylation requires binding of ligands to the PPARγ LBD, resulting in conformational changes that interfere with the ability of CDK5/ERK to phosphorylate Ser-273 [20]. Most of these experiments were performed with murine cells and so far data from primary human white adipocytes are lacking. In addition, it has been proposed that SUs bind to the PPARγ LBD [8,12,13,15] but their impact on PPARγ phosphorylation remains elusive.

In this study, we investigated the binding characteristics of the widely prescribed SUs glibenclamide and glimepiride, representative of a conventional (old) and a modern SU, respectively, to PPARγ focusing on the question whether SUs inhibit PPARγ Ser-273 phosphorylation in comparison to the PPARγ ligands rosiglitazone and SR1664. We found that SUs interfere with PPARγ Ser-273 phosphorylation in the nanomolar range. Despite their low PPARγ transcriptional activity, SUs still elicited an antidiabetic impact on primary human white adipocytes comparable to SR1664. In high-fat diet (HFD) obese mice short-term SU treatment reduced PPARγ Ser-273 phosphorylation in white adipose tissue (WAT) resulting in increased adipogene expression and mRNA reduction of the adipose tissue macrophage marker *F4/80*. In brown adipose tissue (BAT), SUs induced uncoupling protein 1 (UCP1) expression to a similar extend as rosiglitazone but without increasing adipogenesis and BAT weight. Our findings indicate that extra-pancreatic effects frequently observed in patients treated with SUs are probably at least in part driven by inhibition of PPARγ Ser-273 phosphorylation in adipocytes rather than via classical PPARγ agonistic activity.

## 2 Material and methods

### 2.1 Animals

8-week-old male C57Bl/6N mice were fed a HFD (60% energy from fat; Ssniff HFD: EF D12492, #E15741–347) for 16 weeks. Mice were maintained at the Haus für experimentelle Therapie, University Hospital Bonn, or at the Institute of Pharmacology and Toxicology, University Hospital Bonn, during experiments on a daily cycle of 12 h light (06:00 to 18:00) and 12 h darkness (18:00 to 06:00), at 23 ± 1 °C, and were allowed free access to chow and water. Health status was checked frequently and included determination of body weight, observation of unprovoked behavior and responses to external stimuli, as well as assessment of physical appearance. HFD mice were injected intraperitoneally (i.p.) twice daily with 10 mg/kg glimepiride, glibenclamide, rosiglitazone (all Sigma–Aldrich) dissolved in EtOH 10%, PEG 40%, H_2_O 50% or vehicle alone for 6 days. Studies including pharmacokinetics were performed 1 h after the last dose or after overnight starving. All animal experiments have been approved by the local authority Landesamt für Natur, Umwelt und Verbraucherschutz, NRW, Germany (reference: 84–02.04.2014.A202).

### 2.2 Body composition analysis

Body composition was analyzed using a table Bruker Minispec LF50H [28].

### 2.3 Glucose tolerance test

After the last drug treatment animals were fasted overnight. Eight μl/g body weight of glucose solution (2.5 g/mL) were injected i.p. and glucose was measured at indicated time points post injection. Tail vein was punctured and blood was analyzed with an Accu-Chek Aviva Nano analyzer and dipsticks (Roche).

### 2.4 Serum analysis

Serum was collected by blood centrifugation (2000 g, 10 min at RT) and frozen.

For adiponectin determination, serum was diluted 1:30000 in ELISA buffer and adiponectin concentrations were determined using the Adiponectin Mouse ELISA Kit (Invitrogen) according to the manufacturer's instructions.

To determine serum lipids the Piccolo Lipid Plus Panel was used (ABAXIS Europe GmbH) in conjunction with a Piccolo Xpress analyzing system according to the manufacturer's instructions.

### 2.5 Pharmacokinetic analysis

Serum concentrations of glibenclamide, glimepiride and rosiglitazone were analyzed by liquid chromatography – tandem mass spectrometry. All samples were fortified with 10 ng/mL tolbutamide (Sigma–Aldrich) as internal standard. Respective volumes of mouse serum were precipitated with the threefold volume of acetonitrile (−20 °C) and centrifuged for 10 min at 14,000 g. The supernatant was dried under vacuum conditions at 60 °C and subsequently reconstituted with 0.2% formic acid. For all measurements a QTRAP 6500 triple quadrupole MS (Sciex) coupled to a Nexera UPLC (Shimadzu) was used under positive electrospray ionization (ESI) conditions. The system was equipped with an Accucore C8 (50 × 3 mm, 2.6 μm particle size, Thermo Fisher). Gradient elution was applied over 14 min by using (A) 0.2% formic acid, pH 2.5 and (B) acetonitrile with T_min_/B [%] 0.2/2, 8.0/60, 10.0/100, 12.0/100, 12.1/2, and 14.0/2. For quantification (+) MRM ion transitions were *m/z* 494.1 to 169.0, 491.2 to 126.1, 358.1 to 135.1 for glibenclamide, glimepiride and rosiglitazone, respectively. Collision energies (CE) were set to 35 [eV]. Tolbutamide was determined with *m/z* 271.1 to 155.1 and a CE with 27 [eV].

### 2.6 Immunohistochemistry

Five-micrometer paraffin-embedded gonadal WAT (gWAT) and interscapular BAT (iBAT) sections were hydrated and blocked with 2.5% normal goat serum–PBST (phosphate-buffered saline, 0.1 % Tween-20) for 1 h at room temperature. Primary antibodies (GLUT4, 1:50, Santa Cruz; F4/80, 1:10, Invitrogen; UCP1, 1:500, custom made) were applied overnight at 4 °C. After washing three times with PBST, secondary antibody against rabbit (SignalStain Boost IHC, Cell Signaling) was applied for 1 h at room temperature and developed with the DAB Kit (Vector Laboratories) according to the manufacturer's instructions. WAT sections were counterstained with hematoxylin before dehydration. Standard hematoxylin and eosin (H&E) staining was performed with BAT sections. Sections were mounted with RotiHistokit (Carl Roth). Quantification of lipid droplet sizes in H&E-stained BAT sections was analyzed and calculated using the Adiposoft plugin for Fiji (ImageJ). Large lipid droplets were defined as surface >300 μm^2^. One 20x magnification frame of BAT per mouse was scored.

### 2.7 Differentiation of primary human preadipocytes and murine 3T3-L1 preadipocytes

Human primary preadipocytes prepared from liposuction material were obtained from PromoCell and differentiated as previously described [10]. In brief, cells were expanded in growth medium (PromoCell) and differentiation (day 0) was initiated by switching for three days to differentiation medium (PromoCell). Thereafter, the cells were cultured in nutrition medium (PromoCell) containing glibenclamide, glimepiride, rosiglitazone or SR1664 (all Sigma–Aldrich) dissolved in DMSO until day 21 if not otherwise stated. 3T3-L1 preadipocytes were obtained from the American Type Culture Collection (ATCC) and were cultured in Dulbecco's modified Eagle's medium (DMEM, Invitrogen) and 10% fetal bovine serum. Differentiation was induced as previously reported [29]. Glibenclamide, glimepiride, rosiglitazone or SR1664 were added to culture media from induction of differentiation on (day 0) until day 7 if not otherwise stated. Human (Sigma–Aldrich) or murine (Miltenyi Biotec) tumor necrosis factor α (TNFα) were dissolved in water and added to culture media as indicated.

### 2.8 Oil Red O staining and triglyceride measurement

Lipid accumulation of differentiated adipocytes was determined by Oil red O staining or enzymatic determination of triglyceride content. For Oil red O staining, cells were washed in PBS and fixed with 4 % paraformaldehyde at 4 °C for 1 h. Thereafter 1 mL Oil Red O solution (1.25 mg/mL) was added to the wells and incubated for 1 h at room temperature. Finally, cells were washed three times with aqua dest. and were evaluated by light microscopy.

For determination of the triglyceride content, cells were washed once with PBS and after addition of TX lysis buffer (150 mM NaCl, 0.05% Triton X-100, 10 mM Tris–HCl pH 8), wells were immediately frozen at −80 °C. Cells were thawed on ice, sonicated and resuspended. Triglyceride reagent (Sigma–Aldrich) was added and after incubation for 3 h at room temperature in the dark, absorption at 540 nm was measured against TX lysis buffer and a triglyceride standard. Triglyceride content was normalized to the protein content of the sample.

### 2.9 Western blotting

Proteins from cells and gWAT were extracted with RIPA lysis buffer (150 mM NaCl, 50 mM Tris–HCl pH 7.5, 1% Nonidet P40, 0.25% Na-deoxycholat, 0.1% SDS) containing Complete Protease Inhibitor Cocktail (Roche), 1 mM Na_3_VO_4_, and 10 mM NaF. The protein content was determined with the BCA method. Western blotting was performed as previously described [30] using the following primary antibodies: anti-aP2 (1:1000), anti-PPARγ (1:1000), anti-H1 (1:1000), anti-β-actin-HRP (1:20000; all Santa Cruz Biotechnology), anti-CDK substrate antibody (1:500) to detect phospho-Ser in the consensus motif for CDK substrate proteins (KSPXK) and anti-CDK5 (1:1000; all Cell Signaling Technology). A rabbit polyclonal phospho-specific antibody against human PPARγ2 Ser-273 was custom made by Thermo Fisher Scientific with a synthetic phospho-peptide corresponding to residues surrounding Ser-273 of PPARγ2 (Ac-KTTDKpSPFVIYD-C) coupled to KLH.

### 2.10 RNA extraction and quantitative Real-time-PCR

RNA from cells, gWAT and iBAT were extracted with Trizol (Invitrogen). Reverse transcription was performed using the Transcriptor First Strand cDNA Synthesis Kit (Roche). Quantitative Real-time PCR (qRT-PCR) was performed with SYBR Green (Roche) using a HT7900 instrument (Applied Biosystems). Relative mRNA expression was determined by the ΔΔ−Ct method normalized to human glyceraldehyde 3-phosphate dehydrogenase (*GAPDH*) or murine hypoxanthine guanine phosphoribosyl transferase (*Hprt*). Primer pairs are presented in Table S1.

### 2.11 *In vitro* CDK5/P35 kinase assay

*In vitro* CDK5/P35 kinase assays were performed according to the manufacturer's instructions (Cell Signaling Technology). 0.5 μg of recombinant PPARγ LBD (Cayman Chemicals) or purified histone H1 protein (New England BioLabs) and active CDK5/P35 kinase (Millipore) dissolved in kinase assay buffer were pre-incubated with different concentrations of the compounds for 30 min at room temperature as indicated. Kinase reaction was started by adding ATP to a final concentration of 200 μM for 15 min at 30 °C.

For Western blotting kinase reactions were stopped by the addition of Laemmli buffer and heating for 5 min at 99 °C. Ten μl of the reaction were subjected to Western blotting (see above).

To quantitatively measure kinase activity the amount of adenosine diphosphate (ADP) produced during the kinase reaction was determined with the ADP-Glo kinase assay (Promega). Kinase reaction was stopped and the ADP content determined in a 96-well format according to the manufacturer's instructions. Luminescence was measured with an EnSpire Plate Reader (Perkin Elmer).

### 2.12 Glucose uptake assay

Glucose content in culture media was determined with the Glucose Colorimetric Assay Kit (Cayman Chemicals) according to the manufacturer's protocol. Total glucose uptake was calculated by subtracting obtained glucose concentrations of supernatants of treated cells from glucose content of native culture medium.

### 2.13 *In silico* modelling

*In silico* analysis (i.e., ligand docking and structural alignment/comparison) was performed with pre-determined ligand bound crystal structures as well as modelled structures of PPARγ. The details of the methods used are presented in Supplementary Materials.

### 2.14 Luciferase assay

Luciferase assays were performed according to a published protocol using the Dual-Luciferase Reporter Assay System (Promega) [31]. In brief, HEK-293T cells were transfected using Lipofectamine 2000 (Invitrogen) with plasmids containing PPARγ2 (pBabe bleo human PPARγ2), RXRα (pSV Sport RXRα), PPRE (DR1X3) firefly luciferase reporter (PPRE X3-TK-luc) and renilla luciferase (pRL-TK-Renilla, Promega). PPRE X3-TK-luc (Addgene plasmid #1015) and pSV Sport RXRα (Addgene plasmid #8882) were a kind gift from Bruce Spiegelman [32,33]. pBabe bleo human PPARγ2 (Addgene plasmid #11439) was a kind gift from Ronald Kahn. Following an overnight transfection, cells were treated with compounds as indicated for 24 h.

### 2.15 Statistical analysis

Concentration effect curves were fitted to data points by nonlinear regression analysis using the four-parameter logistic equation using Prism software (GraphPad). Statistical significance was determined using one-way or two-way ANOVA analysis of variance with Dunnett's or Tukey's post-hoc tests to compare differences among multiple groups as indicated.

## 3 Results

### 3.1 Transcriptional activity, induction of adipogenesis and suppression of cytokine expression by SUs, rosiglitazone and SR1664 in primary human white adipocytes

**Figure 1:**
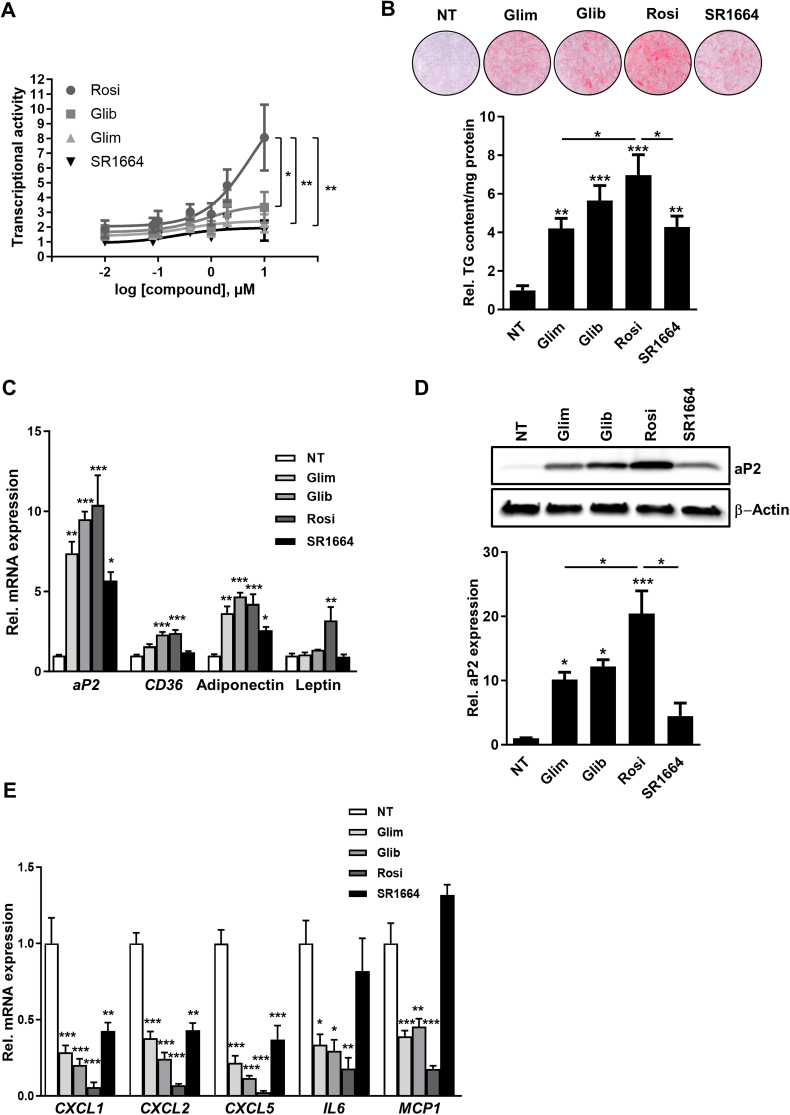
**Transcriptional activity of sulfonylureas, rosiglitazone and SR1664, and effects on primary human white preadipocytes during differentiation.** (**A**) Transcriptional activity (luciferase assays) of a PPAR response element (PPRE) in HEK293T cells following treatment with increasing concentrations (0.01–10 μM) of glimepiride (Glim), glibenclamide (Glib), rosiglitazone (Rosi) and SR1664 (n = 4). Primary human white preadipocytes were differentiated in the presence of 2.5 μM Glim, Glib, Rosi and SR1664 until Day 21. (**B**) Red O staining of differentiated adipocytes (upper panel). Triglyceride (TG) content was quantified using an enzymatic assay and normalized to the protein content of the sample (lower graph, n = 4). (**C**) mRNA expression of adipogenic markers *aP2*, adiponectin*, CD36* and leptin was determined by qRT-PCR (n = 5). (**D**) aP2 protein expression was assessed by Western blotting. β-Actin Western blot was performed to control for loading (upper panels). Quantification of relative aP2 expression vs. β-Actin (n = 3, lower graph) (**E**) mRNA expression of pro-inflammatory cytokines *CXCL1*, *CXCL2*, *CXCL5*, *IL6* and *MCP1* was determined by qRT-PCR (n = 5). Data are represented as means +/− SEM. ∗, p ≤ 0.05; ∗∗, p ≤ 0.01; ∗∗∗, p ≤ 0.001 vs NT or as indicated, One-way ANOVA with Dunnett's or Tukey's post-hoc test. NT, not treated.

TZDs such as rosiglitazone are classical PPARγ full agonists and increase expression of adipogenic genes in adipocytes, which rely on both, PPARγ transcriptional activity and PPARγ Ser-273 phosphorylation. Instead, compounds like the PPARγ ligand SR1664 display low PPARγ transcriptional activity but block phosphorylation of PPARγ at Ser-273 thereby modulating transcription of a subset of genes [19,20]. PPARγ as a transcription factor binds to the PPAR response element (PPRE) in the promoters of its target genes to activate expression. In order to directly compare the PPARγ transcriptional activity of SUs to rosiglitazone and SR1664, we performed luciferase reporter assays. Therefore, we transfected HEK293T cells with PPARγ and a PPRE luciferase reporter construct in the presence of different concentrations of the compounds. Both SUs and SR1664 only weakly increased the reporter activity (1.8-, 2.2-, 3.3-fold at 10 μM for SR1664, glimepiride and glibenclamide, respectively) as compared to rosiglitazone (8-fold increase at 10 μM; Figure 1A). We used primary human preadipocytes as a model system to investigate if SUs, despite low PPARγ transcriptional activity, still can have an impact on adipogenesis when applied during differentiation. Glibenclamide significantly increased adipogenesis almost as strong as rosiglitazone as determined by Oil Red O staining and triglyceride quantification. A statistically significant but lower increase in lipid accumulation was observed for glimepiride and SR1664 (Figure 1B). This is in contrast to murine white adipocytes where SR1664 did not induce adipogenesis [20,27]. Expression of adipogenic markers was determined by qRT-PCR and Western blotting. Adiponectin, *aP2* and *CD3*6 mRNA expression was significantly increased by glibenclamide and rosiglitazone. Instead, glimepiride and SR1664 only increased *aP2* and adiponectin mRNA expression, while *CD36* was not affected (Figure 1C). The increase of adiponectin expression after SU treatment is of particular interest as it depends on PPARγ Ser-273 phosphorylation [19,20]. Leptin mRNA was only significantly increased after rosiglitazone treatment showing that strong PPARγ agonist activity is required for its expression in human adipocytes (Figure 1C). In line with the observed lipid accumulation, aP2 protein expression was significantly increased 10- and 12-fold by glimepiride and glibenclamide, respectively, as compared to 20-fold by rosiglitazone (Figure 1D). Although SR1664 affected aP2 protein expression, this increase was not significantly different from the control (4-fold) (Figure 1D). These data show that transcriptional activation of PPARγ by glibenclamide is still sufficient to enhance adipogenic differentiation of primary human white preadipocytes in a similar fashion to rosiglitazone when applied at equimolar concentrations. In line with the weaker PPARγ transcriptional activity (Figure 1A), adipogenic potency of glimepiride was lower as compared to rosiglitazone and glibenclamide.

Obesity and T2DM are characterized by increased levels of pro-inflammatory cytokines and free fatty acids circulating in blood and tissues [34]. TZDs and SUs are able to reduce expression of pro-inflammatory cytokines in human adipocytes [10]. To directly compare the effect of the four compounds on cytokine expression during differentiation, we also performed qRT–PCR analyses. SUs and rosiglitazone suppressed expression of all cytokines tested (Figure 1E). In contrast, SR1664 only diminished mRNA expression of members of the C-X-C motif chemokine ligand (*CXCL)* family but did not significantly affect interleukin 6 (*IL6*) and monocyte chemoattractant protein 1 (*MCP1*) expression (Figure 1E). Thus, modulation of expression of *IL6* and *MCP1* does not seem to depend on PPARγ Ser-273 phosphorylation in primary human white adipocytes.

### 3.2 SUs block PPARγ Ser-273 phosphorylation and counteract TNFα-induced antidiabetic effects *in vitro* in white adipocytes

**Figure 2:**
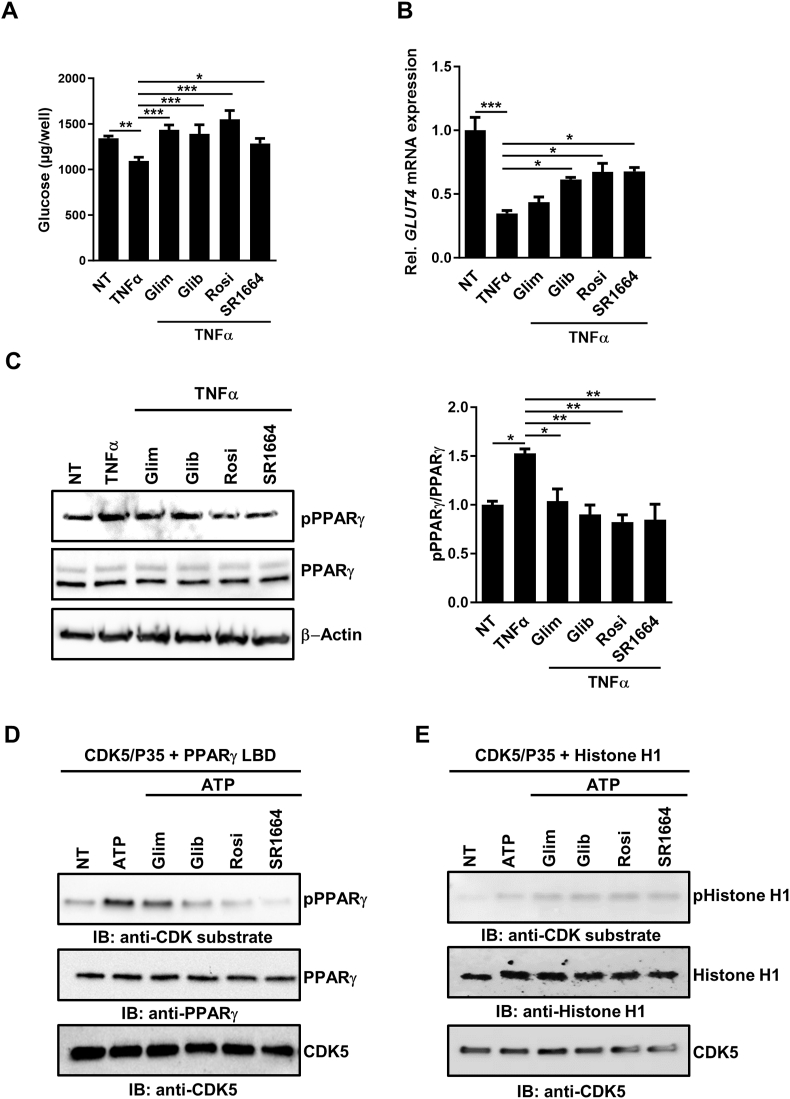
**Sulfonylureas, rosiglitazone and SR1664 have antidiabetic effects and block PPARγ Ser-273 phosphorylation *in vitro*.** (**A**) Total glucose uptake (n = 6) and (**B**) *GLUT4* mRNA expression (n = 3) of differentiated primary human white adipocytes pre-incubated with 2.5 μM glimepiride (Glim), glibenclamide (Glib), rosiglitazone (Rosi) and SR1664 for 45 min prior to treatment with 5 ng/mL human TNFα for 48 h. (**C**) Western blot against phosphorylated PPARγ at Ser-273 (pPPARγ) of differentiated primary human white adipocytes pre-treated with 2.5 μM Glim, Glib, Rosi and SR1664 for 45 min prior to treatment with 5 ng/mL human TNFα for 60 min. PPARγ and β-Actin Western blots were performed to control for loading (left panels). Quantification of relative PPARγ phosphorylation vs. PPARγ expression is shown in the right graph (n = 4). (**D**) *In vitro* CDK5 kinase assay on PPARγ LBD incubated with 2.5 μM Glim, Glib, Rosi and SR1664. Kinase assays were subjected to Western blotting with a CDK substrate specific antibody, PPARγ or CDK5. (**E**) *In vitro* CDK5 kinase assay on Histone H1 incubated with 2.5 μM Glim, Glib, Rosi and SR1664. Kinase assays were subjected to Western blotting with a CDK substrate specific antibody, Histone H1 or CDK5. Data are represented as means +/− SEM. ∗, p ≤ 0.05; ∗∗, p ≤ 0.01; ∗∗∗, p ≤ 0.001 vs TNFα, One-way ANOVA with Dunnett's post-hoc test. IB, immunoblot; NT, not treated.

TNFα induces phosphorylation of PPARγ at Ser-273 in murine adipocytes subsequently promoting insulin resistance [19,20]. To test if TNFα was able to induce insulin resistance in our cellular system and if this can be reverted by SUs as compared to rosiglitazone and SR1664, we measured overall glucose uptake and glucose transporter 4 (*GLUT4*) mRNA expression of primary human white adipocytes after stimulation with TNFα. Preincubation of cells with rosiglitazone completely prevented TNFα-induced reduction of glucose uptake (Figure 2A) and diminished suppression of *GLUT4* mRNA expression (Figure 2B). But, also SUs and SR1664 counteracted the TNFα-induced effects (Figure 2A,B). This shows that glucose trafficking during TNFα-induced diabetic processes in human adipocytes predominantly relies on PPARγ Ser-273 phosphorylation and not on PPARγ agonist activity.

Next, we analyzed the underlying mechanisms in both murine and human white adipocytes. TNFα induced PPARγ Ser-273 phosphorylation in primary human white adipocytes in a concentration-dependent manner as shown by phospho-PPARγ Ser-273 Western blots (Figure S1A). To address if SUs block PPARγ Ser-273 phosphorylation, we pre-incubated primary human white adipocytes with rosiglitazone, SR1664 and SUs prior to stimulation with TNFα. Treatment with all compounds resulted in a significant reduction in TNFα-induced PPARγ phosphorylation, bringing phospho-Ser-273 levels close to baseline (Figure 2C). A similar effect of SUs was also observed in differentiated murine 3T3-L1 adipocytes (Figure S1B).

To exclude indirect effects of SUs on CDK5 kinase activity, we assessed whether SUs modulate PPARγ Ser-273 phosphorylation using a CDK5 kinase assay. SUs reduced phosphorylation of the PPARγ LBD as shown with a CDK5 substrate specific antibody similar to rosiglitazone and SR1664 (Figure 2D). It is noteworthy that this inhibition is not caused by a general inhibition of CDK5 activity, as incubation with the test compounds did not inhibit the ability of CDK5 to phosphorylate its well-known substrate histone H1 (Figure 2E).

### 3.3 Concentration-response relationships of PPARγ Ser-273 phosphorylation

**Figure 3:**
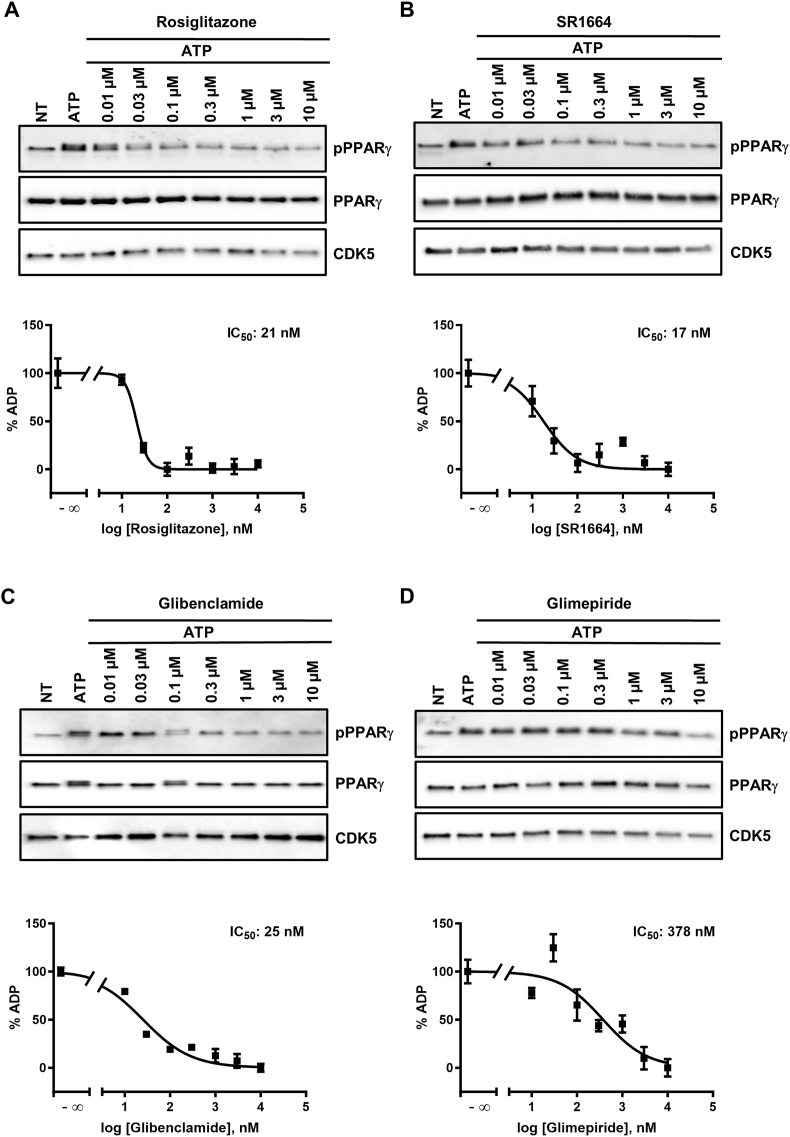
**Sulfonylureas inhibit PPARγ Ser-273 phosphorylation in a concentration-dependent manner *in vitro*.** *In vitro* CDK5 kinase assays on PPARγ LBD incubated with increasing concentrations (0.01–10 μM) of (**A**) rosiglitazone, (**B**) SR1664, (**C**) glibenclamide and (**D**) glimepiride. Kinase reactions were subjected to Western blotting against phosphorylated PPARγ at Ser-273, PPARγ or CDK5 (A-D, upper panels). ADP content of the same kinase reactions was determined by a chemoluminescent assay (A-D, lower graphs). Data are represented as means +/− SEM (n = 4). IC_50_ values were determined from the fitted concentration-response curves. NT, not treated.

To further study the SU-induced effects, we recorded concentration-response relationships of CDK5 kinase inhibition and determined half-maximal inhibitory concentrations (IC_50s_). To this end, we quantitatively measured the amount of ADP produced during the kinase reaction by a luminescent ADP detection assay. Notably, glibenclamide showed a comparable phosphorylation inhibition of the PPARγ LBD to rosiglitazone and SR1664 with IC_50s_ of 25 nM, 21 nM and 17 nM, respectively (Figure 3A–C) while glimepiride displayed an approximately 15-fold lower potency (IC_50_: 378 nM; Figure 3D).

### 3.4 SUs reduce PPARγ Ser-273 phosphorylation in WAT of HFD mice

**Figure 4:**
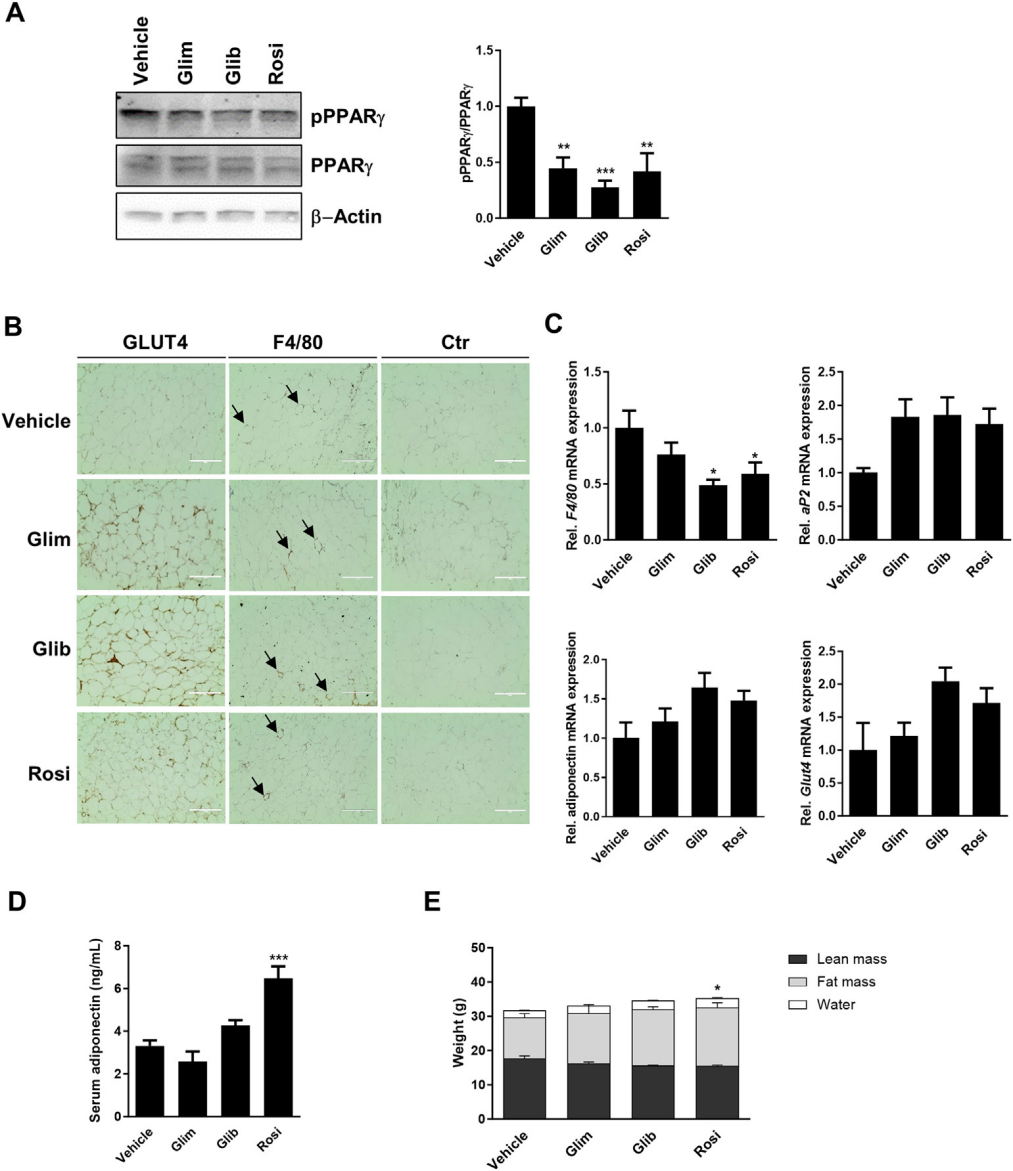
**White adipose tissue (WAT) analysis of high-fat diet (HFD) mice after 6 days treatment with glimepiride (Glim), glibenclamide (Glib) and rosiglitazone (Rosi).** (**A**) Western blot against phosphorylated PPARγ at Ser-273 (pPPARγ). PPARγ and β-Actin Western blots were performed to control for loading (left), quantification of relative PPARγ phosphorylation vs. PPARγ expression (right). (**B**) Immunohistochemistry of GLUT4 and F4/80 expression in WAT, crown-like structures are marked with an arrow. Control slides (Ctr) were stained with hematoxylin and secondary antibodies; scale bar 200 μm. (**C**) Relative *F4/80*, *aP2*, adiponectin and *Glut4* mRNA expression in WAT was determined by qRT-PCR. (**D**) Adiponectin serum levels and (**E**) body composition of HFD mice. Data are represented as means +/− SEM (n = 5). ∗, p ≤ 0.05; ∗∗, p ≤ 0.01; ∗∗∗, p ≤ 0.001 vs Vehicle, One-way ANOVA with Dunnett's post-hoc test.

During diet-induced obesity, mice develop insulin resistance and exhibit increased PPARγ Ser-273 phosphorylation in WAT [19]. In order to assess *in vivo* relevance of our findings, we investigated if SUs also block PPARγ phosphorylation in WAT of HFD mice. After treatment for 6 days with a twice-daily i.p. dose of 10 mg/kg we determined mean plasma concentrations of 5 µM, 7 μM and 20 μM for glimepiride, glibenclamide and rosiglitazone, respectively (Figure S2A). All drugs caused a similar decrease in the phosphorylation of PPARγ Ser-273 in WAT (Figure 4A) and an increase in GLUT4 protein expression as demonstrated by immunohistochemistry (Figure 4B). Although we did not observe a treatment-related reduction in crown-like structures in WAT stained with the macrophage marker F4/80 as signs of macrophage infiltration (Figure 4B), *F4/*80 mRNA expression was reduced by 24% for glimepiride (not significant) and about 50% for glibenclamide and rosiglitazone (Figure 4C). There was also increased expression of *aP2*, adiponectin and *Glut4* mRNA in WAT of all treatment groups albeit this did not reach statistical significance (Figure 4C). In line with the increase in mRNA abundance, we measured slightly increased adiponectin serum levels in glibenclamide-treated mice. A statistical significant 2-fold increase was induced by rosiglitazone while glimepiride had no effect (Figure 4D). Only rosiglitazone induced minor changes in body composition (slightly increased fat and water mass; Figure 4E), but there were no effects on blood glucose, serum triglycerides and cholesterol levels by any of the drugs (Figure S2B–E). When we starved mice overnight, SU-treated mice exhibited a paradox increase in blood glucose and reduced glucose tolerance while rosiglitazone reduced blood glucose as expected (Figure S2B and F). This phenomenon might be the result of counterregulatory mechanisms after chronic SU treatment and overnight starving [35,36].

### 3.5 SUs alter adipocyte morphology and thermogenic marker expression in BAT of HFD mice

**Figure 5:**
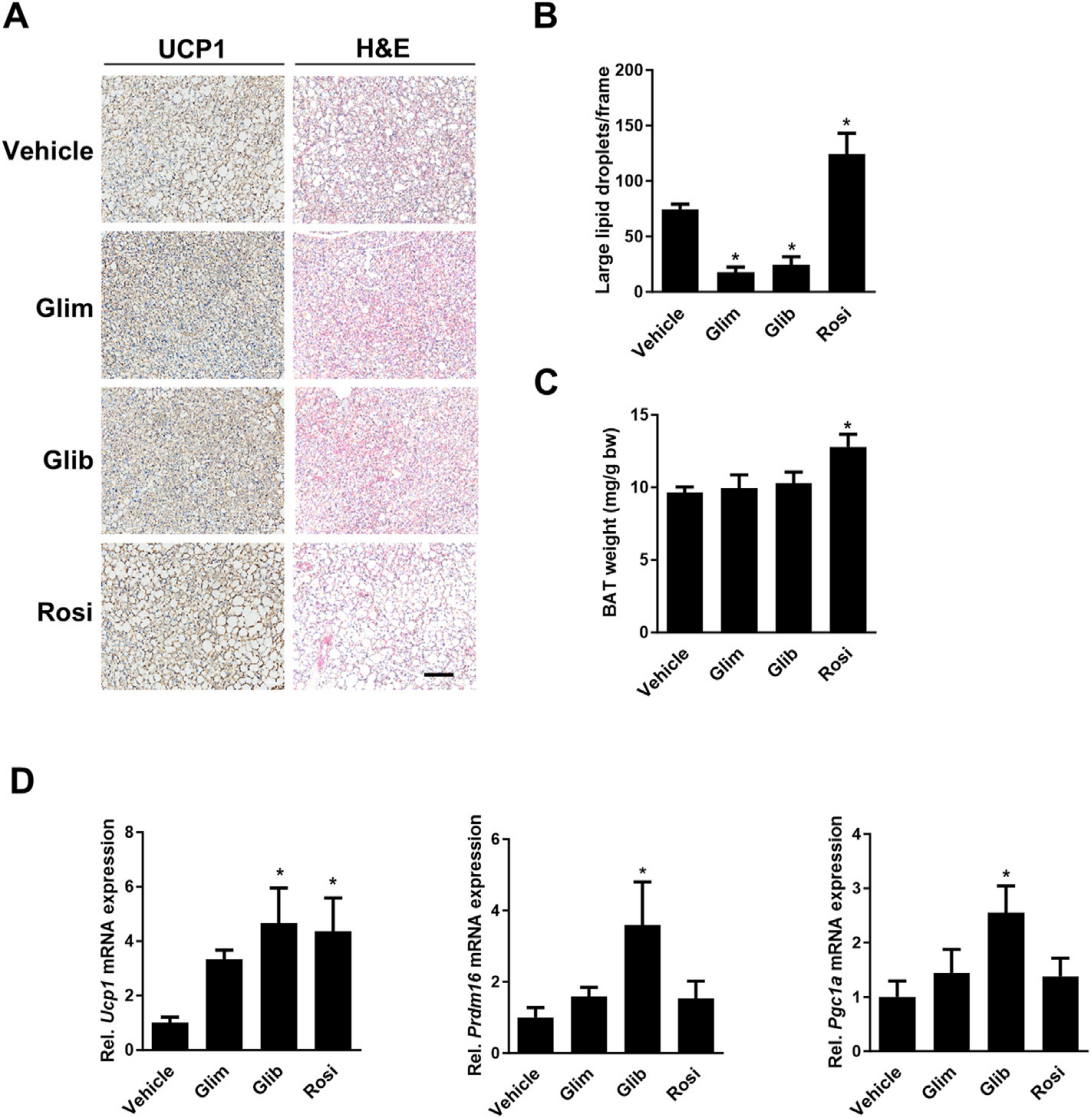
**Brown adipose tissue (BAT) analysis of high-fat diet (HFD) mice after 6 days treatment with glimepiride (Glim), glibenclamide (Glib) and rosiglitazone (Rosi).** (**A**) Hematoxylin/eosin (H&E) staining (right panels) and immunohistochemistry of UCP1 expression in BAT (left panels); scale bar 100 μm. (**B**) Quantification of lipid droplet sizes per 20x magnification frame/mouse. Large lipid droplets were defined as surface >300 μm^2^. (**C**) BAT weight per g body weight (bw). (**D**) Relative *Ucp1*, *Prdm16* and *Pgc1a* mRNA expression was determined by qRT-PCR in BAT. Data are represented as means +/− SEM (n = 5). ∗, p ≤ 0.05 vs Vehicle, One-way ANOVA with Dunnett's post-hoc test.

Glibenclamide and some PPARγ Ser-273 blockers can induce thermogenic gene expression in BAT and impact brown adipocyte morphology [[37], [38], [39], [40], [41], [42]]. Therefore, we also performed a histological analysis of BAT. As expected [43], H&E staining of BAT of rosiglitazone-treated mice was characterized by larger lipid droplets and significantly increased weight (+33%) as compared to BAT from control mice (Figure 5A–C). In contrast, SU treatment resulted in much smaller lipid droplets with no significant changes in BAT mass (Figure 5A–C). These changes were accompanied by increased UCP1 expression as determined by immunohistochemistry and qRT–PCR analyses. While visual changes in UCP1 protein expression were small in the immunohistochemistry (Figure 5A), *Ucp1* mRNA increased by 3.3-, 4.7- and 4.4-fold after treatment with glimepiride, glibenclamide and rosiglitazone, respectively (Figure 5D). The mRNAs of two other thermogenic markers, PR domain containing 16 (*Prdm16*) and Peroxisome proliferator-activated receptor gamma coactivator 1 α (*Pgc1a*), were only significantly increased by glibenclamide.

### 3.6 *In silico* modelling of ligand binding to PPARγ

**Figure 6:**
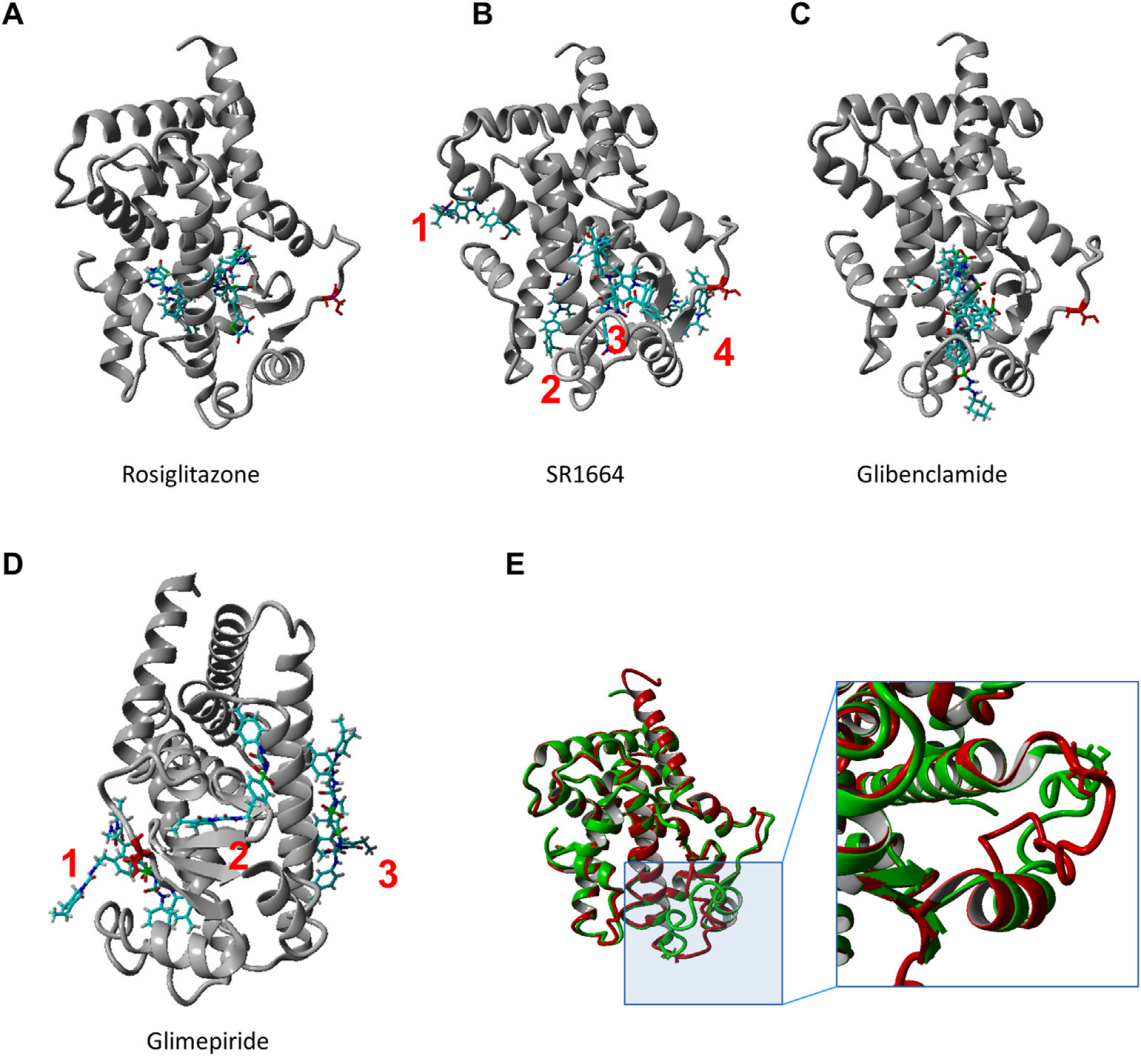
**Docking of ligands onto PPARγ structure and structural alignment of bound (2hfp) and unbound (model) ligand binding regions.** Top 5 docking poses for (**A**) rosiglitazone, (**B**) SR1664, (**C**) glibenclamide and (**D**) glimepiride. In each panel, the protein backbone is depicted in grey colored ribbon format with the ligand shown as stick models colored atom wise. The Ser-273 residue is depicted in each panel as a red colored stick model depiction. In case of docking poses belonging to different regions/ensembles they have been numbered. (**E**) Structural alignment bound (2hfp; green ribbon depiction) and unbound (model; red ribbon depiction) ligand binding regions. The inset image shows a closer view of the loop bearing the Ser-273 residue. The Ser-273 residue is depicted in stick model format.

To gain a structural knowledge of how SUs bind to PPARγ in comparison to rosiglitazone and SR1664, we used an *in silico* modelling approach. We found eight different crystal structures of PPARγ bound to rosiglitazone and two bound to SR1664 in the Protein Data Bank (PDB). Multiple structural alignments of those crystal structures showed that both ligands occupied similar positions. Rosiglitazone occupied the core of the PPARγ LBD interacting with multiple residues on two helixes surrounding this core region as well as on a β-stranded region that connects two helixes and is surrounded on the periphery by a loop that bears the Ser-273 residue (Figure S3A). SR1664 also occupies a similar position but like rosiglitazone, it has no direct contacts with the Ser-273 residue. Instead, it interacts with the β-stranded region proximal to this critical residue (Figure S3B). Although the Ser-273 residue is located on a flexible region, it does not show any major shift (everything <1 Å) in backbone in the different conformers of all these ligand bound complexes. The docking of rosiglitazone, SR1664 and both SUs onto a ligand-bound PPARγ crystal structure (2hfp) [44] from which the ligand was manually removed, suggested the following binding characteristics: A) The top 5 docks for rosiglitazone (Figure 6A) and SR1664 (Figure 6B) were within 1 Å of the region observed for these ligands in their crystal structures validating our docking results. B) The top 5 docks for rosiglitazone and glibenclamide (Figure 6A,C) all converged onto one particular ensemble which was within the core of the PPARγ LBD. C) The top 5 docks for glimepiride (Figure 6D) on the other hand belong to three separate ensembles, all of which have mostly surface presence i.e. none of them are located deep in the core of the LBD. Only one of these ensembles (number 1 in Figure 6D) is partially embedded and more interestingly directly interacts with the loop region bearing the Ser-273 residue where it might interfere with Ser-273 phosphorylation. D) Three of 5 top docking ensembles of SR1664 were surface bound with one of them (number 4 in Figure 6B) close to the loop on which the Ser-273 residue resides. One of the ensembles corresponds to the original location of SR1664 observed in reported crystal structures i.e. the core of the PPARγ LBD (number 3 in Figure 6B). Therefore, it seems that SR1664 shows binding modes that are similar to both glibenclamide/rosiglitazone and glimepiride. In a next step, we compared a native unbound PPARγ structure generated by the I-TASSER threading server to the ligand-bound structures. However, using the complete PPARγ protein yielded very poor scores (C score: 2-23, Figure S4). We observed that this was owed to lack of adequate threading templates for the DNA-binding region. The ligand-binding region within the model showed excellent alignment with various threaded templates and root mean squared deviation (RMSD) between 0.8 and 1.5 Å for the top ten templates. Therefore, for comparison we only considered the ligand-binding region i.e. between residues 230–515 from the model. When we aligned the model to the ligand-bound (i.e. rosiglitazone and SR1664) structures of PPARγ we observed that almost the entire protein except for the loop region containing Ser-273 aligns perfectly. This loop region shows a drastic conformational change when compared to the same region within the unbound structure with an average RMSD of residues from this region >4 Å (Figure 6E), which could interfere with phosphorylation of the Ser-273 residue.

## 4 Discussion

Transcriptional activation of PPARγ in white adipocytes has been suggested as extra-pancreatic mechanism by which SUs increase insulin sensitivity and reduce cytokine expression a part from their classical action on pancreatic β-cells [[8], [9], [10],^12^,^13^,^15^]. However, it was questioned whether these effects on adipocytes are indeed mediated via classical transcriptional activation of PPARγ or alternative mechanisms like interference with PPARγ phosphorylation as described for several PPARγ ligands [45]. Inhibition of PPARγ Ser-273 phosphorylation by PPARγ ligands requires binding to the LBD [19]. Although binding of SUs to the LBD has been proposed by several groups [8,12,13,15], it was unclear so far whether SUs also interfere with Ser-273 phosphorylation. Here, we show that glibenclamide and glimepiride exert similar effects on PPARγ Ser-273 phosphorylation as the full PPARγ agonist rosiglitazone and the PPARγ ligand SR1664 in *in vitro* kinase assays. While glibenclamide was equally potent as rosiglitazone and SR1664 (IC_50s_: 25 nM, 21 nM, and 17 nM, respectively), glimepiride had a 15-fold lower potency (IC_50_: 378 nM) on phosphorylation inhibition. Our *in silico* PPARγ binding analysis revealed that the mode of action of glibenclamide is quite similar to rosiglitazone. Both bind to the core of the protein (i.e. the LBD) inducing a conformational change that allosterically alters the protein surface (i.e. the Ser-273 residue). This mode of action was not observed for glimepiride as it does not bind to the LBD. On the other hand, glimepiride might directly interfere with the protein surface and the Ser-273 residue, however, no docking pose was partially favoured in the docking analysis. Therefore, this binding is probably of low affinity which might account for its lower potency observed in kinase assays.

In patients, the maximum plasma concentrations of glibenclamide and glimepiride are around 1 μM [46,47]. Thus, also clinical concentrations should cause an inhibition of PPARγ Ser-273 phosphorylation in adipocytes. Applying glibenclamide and glimepiride at concentrations close to the clinical range (2.5 μM) was sufficient to suppress TNF-α-induced Ser-273 phosphorylation in primary human adipocytes. Consequently, we observed modulation of Ser-273-related genes such as up regulation of adiponectin and down regulation of pro-inflammatory cytokines similar to SR1664. However, in contrast to SUs and rosiglitazone, SR1664 did not suppress expression of *MCP1* and *IL6*. Similar findings have been reported for murine 3T3-L1 adipocytes [27]. Thus, glibenclamide and glimepiride mediate a distinct PPARγ transcriptional activation, which is required to modulate expression of *MCP1* and *IL6*.

Short-term treatment of HFD mice reduced phosphorylation of PPARγ Ser-273 in WAT by SUs comparable to rosiglitazone. However, we did not observe strong effects of the drugs on body composition, serum chemistry, adipogenic marker expression and adipose tissue inflammation in line with previous short-term studies performed with TZDs [19,20]. Nevertheless, rosiglitazone improved glucose tolerance. SUs induced a paradox effect, which was characterized by increased fasting plasma glucose levels and a negative impact on glucose tolerance. This phenomenon has previously been described after subacute and chronic SU-treatment of mice [35,36]. β-cell desensitization has been suggested as underlying mechanism resulting in an impairment of insulin secretion. In patients, these paradox effects are usually only observed after much longer treatment periods [35,36]. Therefore, longer treatment of mice with SUs was not considered in our study.

It has previously been shown that glimepiride can induce GLUT4 expression in WAT of *SUR1*-deficient HFD rats without elevating insulin, indicating that glimepiride acts via an extra-pancreatic mechanism [48]. In line with this, both SUs also increased GLUT4 protein levels in WAT of HFD mice. Furthermore, in our model of (TNFα-induced) insulin-resistant primary human white adipocytes SUs and SR1664 likewise increased *GLUT4* expression and glucose uptake. Thus, down-regulation of GLUT4 expression and reduced glucose uptake in white adipocytes during diabetic processes might be linked to PPARγ Ser-273 phosphorylation and could be counteracted by SUs.

Several PPARγ Ser-273 blocker reduce adipogenesis in BAT and WAT while increasing thermogenic gene expression and energy expenditure [[38], [39], [40], [41], [42]]. This is in contrast to classical TZDs, which induce adipogenesis leading to weight gain despite increased energy expenditure. This is somehow surprising because the induction of a thermogenic gene program by PPARγ ligands has been linked to full PPARγ agonism [43]. One explanation might be that some PPARγ ligands still have remaining PPARγ transcriptional activity, which is sufficient to induce thermogenic gene expression. On the other hand, the NAD-dependent deacetylase SirT1 is able to deacetylate PPARγ at Lys-268 and Lys-293 thereby driving TZD-induced browning of WAT [49,50]. Considering that the Ser-273 residue is in close proximity to Lys-268 and Lys-293, some PPARγ ligands might also interfere with PPARγ deacetylation to induce thermogenic genes. Recently, increased thermogenesis and UCP1 expression has also been reported after glibenclamide treatment of HFD mice [37]. Similarly, we observed an induction of UCP1 expression in BAT and a reduction in lipid droplet sizes after SU treatment indicative of increased thermogenesis resembling the typical phenotype reported for many PPARγ Ser-273 blocker [[37], [38], [39], [40], [41], [42]]. Although we noted adipogenic effects of SUs in white adipocytes, this did not translate into increased adipogenesis in BAT as observed for rosiglitazone. Therefore, we assume that the SU-induced BAT phenotype is not related to PPARγ agonist activity but might rather depend on PPARγ Ser-273 phosphorylation or alternative mechanisms like interference with PPARγ deacetylation.

In summary, we found that both SUs are able to inhibit PPARγ Ser-273 phosphorylation in primary human white adipocytes *in vitro* and in obese mice. Although we did not directly show that inhibition of PPARγ phosphorylation is linked to the observed antidiabetic effects we provide indirect evidence. For instance, both SUs modulate expression of adipokines and increase GLUT4 expression and glucose uptake similar to SR1664, which exclusively acts by blocking PPARγ Ser-273 phosphorylation. In addition, the observed BAT phenotype was similar to what is observed after treatment of obese mice with some PPARγ Ser-273 blockers and different to rosiglitazone. Glibenclamide was more potent in terms of phosphorylation inhibition in kinase assays and we also observed a higher PPARγ transcriptional and adipogenic activity as compared to glimepiride. In this context it is noteworthy that T2DM patients treated with glibenclamide are at a higher risk of cardiovascular adverse events and weight gain, common side effects also observed with TZDs [51]. This could be due to the observed PPARγ agonist and resulting adipogenic activity despite the beneficial blocking of PPARγ Ser-273 phosphorylation. In line with its rather weak adipogenic activity, glimepiride treatment is associated with fewer of these side effects. Furthermore, glimepiride increases insulin sensitivity and reduces pro-inflammatory cytokines in patients [51]. This might be a result of its interference with PPARγ Ser-273 phosphorylation. Studies of the PPARγ Ser-273 phosphorylation status in tissues of patients treated with SUs could give insight if relevant inhibition can indeed be achieved in humans at clinically relevant doses.

## 5 Conclusion

Our data presented here propose a novel mode of action of SUs on adipocytes, which could help to further explain the insulin-sensitizing and anti-inflammatory effects of SUs observed in the clinic. A portion of this action might be mediated via their interference with PPARγ Ser-273 phosphorylation rather than via classical agonistic activity in adipocytes.

## Funding

This work was supported by an intramural funding of the Federal Institute for Drugs and Medical Devices (to B.H.) and by Deutsche Forschungsgemeinschaft (DFG, German Research Foundation) 450149205-TRR333 (to A.P.). The funders had no role in study design, data collection and analysis, decision to publish, or preparation of the manuscript.

## CRediT authorship contribution statement

**Bodo Haas:** Conceptualization, Formal analysis, Investigation, Supervision, Visualization, Writing – original draft. **Moritz David Sebastian Hass:** Formal analysis, Investigation, Writing - Original Draft, Visualization. **Alexander Voltz:** Investigation, Visualization. **Matthias Vogel:** Investigation, Visualization. **Julia Walther:** Investigation, Visualization. **Arijit Biswas:** Formal analysis, Investigation, Visualization, Writing – original draft. **Daniela Hass:** Investigation, Visualization. **Alexander Pfeifer:** Supervision, Writing – review & editing.

## Declaration of competing interest

Authors declare that there are no conflicts of interests.

## Data Availability

Data will be made available on request.
