# Supplementary material for: Sulfonylureas exert antidiabetic action on adipocytes by inhibition of PPARγ serine 273 phosphorylation
Source: Mol Metab. 2024 May 10;85:101956. doi: 10.1016/j.molmet.2024.101956 (PMC11112612; doi:10.1016/j.molmet.2024.101956)
Supplement: Multimedia component 1 [file mmc1.docx]

**Supplementary Material**

Manuscript title:

**Sulfonylureas exert antidiabetic action on adipocytes by inhibition of PPARγ serine 273 phosphorylation**

Authors:

Bodo Haas^1^, Moritz David Sebastian Hass^1,2^, Alexander Voltz^3^, Matthias Vogel^1^, Julia Walther^1^, Arijit Biswas^4^, Daniela Hass^3,5^, Alexander Pfeifer^3^

Contents:

**Supplementary Figures 1-4**

**Supplementary Figure legends**

**Supplementary Methods**

**Supplementary Table 1**


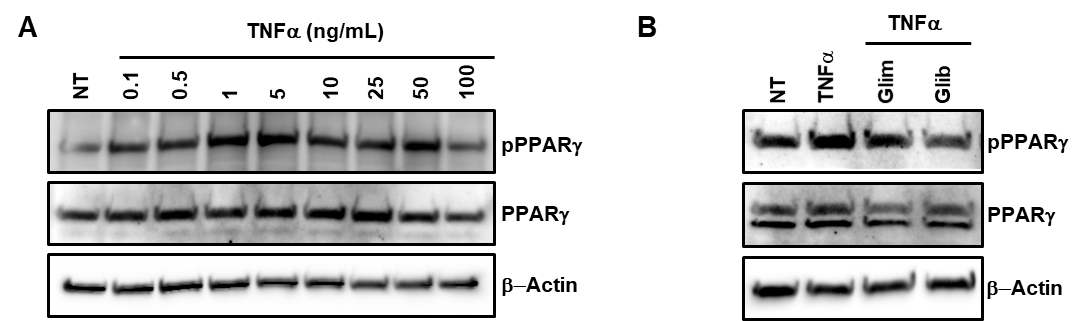


**B**


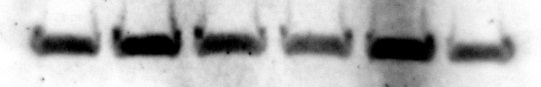

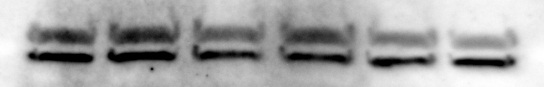

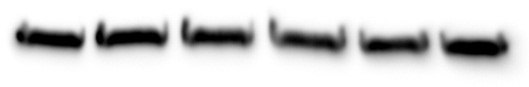


**Glib**

**Glim**

**NT**

**TNFα **

**Ctr**

**pPPARγ**

**PPARγ**

**β−Actin**

**Figure S1.** (**A**) Western blot against phosphorylated PPARγ at Ser-273 of differentiated primary human white adipocytes treated with increasing concentrations of human TNFα for 60 minutes. PPARγ and β-Actin Western blots were performed to control for loading. (**B**) Western blot against phosphorylated PPARγ at Ser-273 of differentiated murine 3T3-L1 adipocytes pre-treated with 2.5 µM glimepiride (Glim) and glibenclamide (Glib) for 45 minutes before stimulation with 5 ng/mL murine TNFα for 60 minutes. PPARγ and β-Actin Western blots were performed to control for loading. NT, not treated.

**
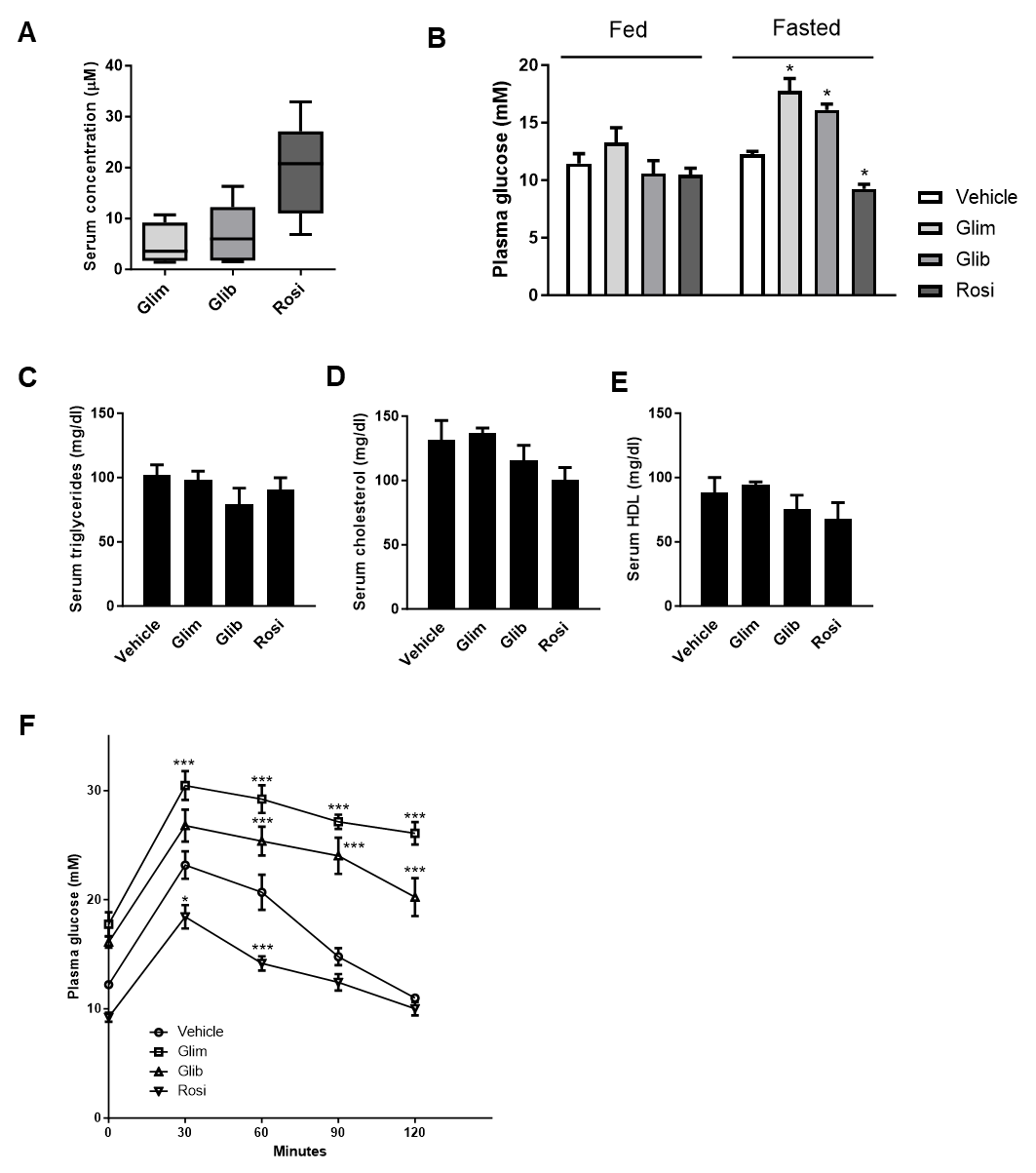
**

**Figure S2.** (**A**) Serum concentrations of glimepiride (Glim), glibenclamide (Glib) and rosiglitazone (Rosi) in HFD mice 1 h after the last administration at day 6 were determined by LC-MS analysis (n = 4-6). (**B**) Plasma glucose of HFD mice was determined in the fed state or after an overnight fast. (**C**) Serum triglycerides, (**D**) cholesterol and (**E**) high density lipoprotein (HDL) levels of HFD mice. (**F**) Glucose tolerance test of HFD mice after an overnight fast. Data are represented as means +/- SEM (n = 3-5). *, p ≤ 0.05; **, p ≤ 0.01; ***, p ≤ 0.001 vs Vehicle, One-way ANOVA with Dunnett’s post-hoc test for B-E, Two-way ANOVA with Dunnett’s post-hoc test for F.


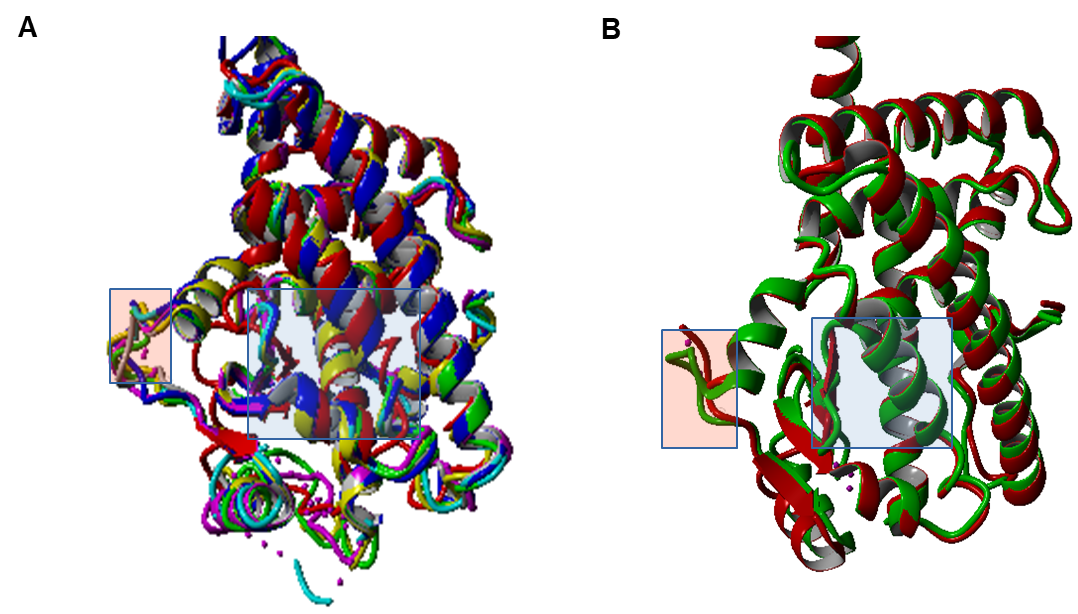


**Figure S3.** Structural alignment of eight human PPARγ structures bound to rosiglitazone (**A**) and two human PPARγ structures bound to SR1664 (**B**). The protein backbone is depicted in ribbon format with different colors for the different structures. The regions occupied by the ligand and Ser-273 residue are depicted by blue and red shaded regions respectively.


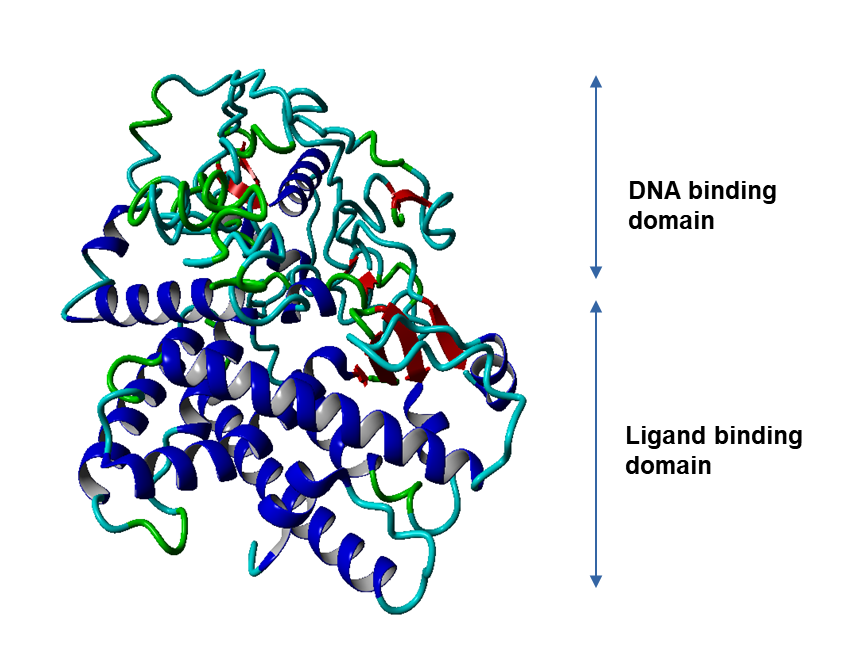


**Figure S4.** Best model of PPARγ generated from the I-TASSER threading and modelling server. The protein backbone is depicted in ribbon format and colored based on secondary structure.

**Supplementary Methods**

***In silico* modelling**

All structures related to human PPARγ protein available in the Protein Data Bank (PDB) were downloaded and analysed for the relative ligand/receptor position using multiple structural alignment. We found eight different crystal structures (PDB IDs: 1FM6, 1ZGY, 2PRG, 3CS8, 3DZY, 4EMA, 4O8F and 4XLD) of PPARγ bound to rosiglitazone (Ligand ID: BRL) and two bound (PDB IDs: 4R2U and 5DWL) to SR1664 (Ligand ID: 3JX) in the PDB. Another PPARγ crystal structure (PDB DOI: https://doi.org/10.2210/pdb2HFP/pdb) 2hfp (resolution: 2Å) but in complex with N-sulfonyl-2-indole carboxamide was chosen to conduct further docking exercises [1]. All heteroatoms as well as water molecules within the structure (2hfp) were manually removed. The structure was subjected to MD refinement simulation as per previously established protocols in order to remove any clashes and bumps incorporated within the structure by static crystallization/ligand binding forces [2]. The lowest energy structure within this simulation trajectory was chosen for docking ligands. Ligand structures pertaining to glibenclamide (PubChem CID: 3488), glimepiride (PubChem CID: 3476), rosiglitazone (PubChem CID: 77999) and SR1664 (PubChem CID: 50919249) were downloaded in SDF format from the pubchem database (https://pubchem.ncbi.nlm.nih.gov/; accessed on 12.08.2016) and converted to the PDB format on YASARA version 13.1.12. All charges were assigned using the AUTOSMILES function embedded in YASARA. Flexible ensemble docking (both receptor and ligand are treated flexibly) was performed with the VINA docking algorithm embedded within YASARA. The top 5 docking positions based on pseudo-binding energy scores were analysed for each ligand docking exercise. Since we could not find a single PPARγ structure that was not bound by ligand we generated a model for the same on the ITASSER threading server in order to compare the bound protein to the unbound one [3]. All structural analysis, visualization and image rendering were performed on YASARA version 13.1.12 [4].

**References**

1. Hopkins C.R., O'neil S.V., Laufersweiler M.C. et al. Design and synthesis of novel N-sulfonyl-2-indole carboxamides as potent PPAR-gamma binding agents with potential application to the treatment of osteoporosis. *Bioorg Med Chem Lett* 2006; **16**:5659-5663.

2. Krieger E., Koraimann G., Vriend G. Increasing the precision of comparative models with YASARA NOVA--a self-parameterizing force field. *Proteins* 2002; **47**:393-402.

3. Yang J., Yan R., Roy A., Xu D., Poisson J., Zhang Y. The I-TASSER Suite: protein structure and function prediction. *Nat Methods* 2015; **12**:7-8.

4. Krieger E., Vriend G. YASARA View - molecular graphics for all devices - from smartphones to workstations. *Bioinformatics* 2014; **30**:2981-2982.

**Table S1.** PCR primers used for quantitative Real-time PCR

| **Gene name** | **Species** | **Upstream primer (5’→3’)** | **Downstream primer (5’→3’)** |
| --- | --- | --- | --- |
| Adiponectin | human | GGCCATCTCCTCCTCACTTCCATTCT | TCCGGTTTCACCGATGTCTCCCTTA |
| *aP2 (FABP4)* | human | TCATACTGGGCCAGGAATTTGACGA | ATGCGAACTTCAGTCCAGGTCAACG |
| *CCL2 (MCP1)* | human | AATTCTCAAACTGAAGCTCGCACTCTCG | CTTGGGTTGTGGAGTGAGTGTTCAAGTCT |
| *CD36* | human | AAATGGGCTGTGACCGGAACTGTG | GGGCTGCAGGAAAGAGACTGTGTTGT |
| *CXCL1* | human | CATGGCCCGCGCTGCTCTCT | CTCCCTTCTGGTCAGTTGGATTTGTCAC |
| *CXCL2* | human | AACCGCCTGCTGAGCCCCAT | AAGCTTCCTCCTTCCTTCTGGTCAGTTG |
| *CXCL5* | human | CTCGCAGCGCTCTCTTGACCACTAT | TCCATGCGTGCTCATTTCTCTTAATCAG |
| *GLUT4* | human | AGCTCTCTGGCATCAATGCTGTTTTCTA | GAAGTTGCTCGTCCAGTTGGAGAAACC |
| *GAPDH* | human | TCCTGTTCGACAGTCAGCCGCAT | TGAAGACGCCAGTGGACTCCACG |
| *IL6* | human | CCC CCA GGA GAA GAT TCC AAA GAT GTA G | GTG GTT GGG TCA GGG GTG GTT ATT G |
| Leptin | human | CTATGTCCAAGCTGTGCCCATCCAA | GAGTGACCTTCAAGGCCTCAGCACC |
| *PGC1a* | human | ATGGCGTGGGACATGTGCAACC | AGGGACGTCTTTGTGGCTTTTGCTG |
| Adiponectin | mouse | GCCGTTCTCTTCACCTACGA | CATACACCTGGAGCCAGACTT |
| *aP2 (fabp4)* | mouse | TGA AAG AAG TGG GAG TGG GCT TTG C | CAC CAC CAG CTT GTC ACC ATC TCG T |
| *F4/80* | mouse | TATGCCACCTGCACTGACAC | GCAGACTGAGTTAGGACCACAA |
| *Glut4* | mouse | CTTATTGCAGCGCCTGAGTC | TCCCCATCCTTACGTCAGAGC |
| *Hprt* | mouse | GTCCCAGCGTCGTCGTGATTAGC | TCATGACATCTCGAGCAAGTCTTT |
| *Pgc1a* | mouse | TGT GGA ACT CTC TGG AAC TGC | AGG GTT ATC TTG GTT GGC TTT A |
| *Prdm16* | mouse | AGG TGT CAT CCC AGG AGA GCT GCA T | TAC TTG TTG GGG AAC ATC CGC TCG |
| *Ucp1* | mouse | GGC CTC TAG GAC TCA GTC | TAA GCC GGC TGA GAT CTT GT |
